# The characterization of the physicochemical and sensory properties of full-fat, reduced-fat and low-fat bovine, caprine, and ovine Greek yogurt (Labneh)

**DOI:** 10.1002/fsn3.89

**Published:** 2014-02-07

**Authors:** Samson Atamian, Ammar Olabi, Omar Kebbe Baghdadi, Imad Toufeili

**Affiliations:** 1 Nutrition and Food Science Department, American University of BeirutRiad El Solh, 1107 2020, Beirut, Lebanon

**Keywords:** Caprine, concentrated yogurt, Labneh, low-fat, ovine, sensory

## Abstract

Concentrated/Greek yogurt or Labneh is a semisolid food produced from yogurt by eliminating part of its water and water-soluble compounds. Today's world is geared toward the production of lower fat foods without compromising the texture and flavor of these products. The objective of this study was to characterize the physicochemical and sensory properties of bovine, caprine, and ovine Labneh with different fat levels. Bovine, caprine, and ovine milks were used to produce two batches of full-fat (∼10%), reduced-fat (∼5%), and low-fat (<1%) concentrated yogurt samples. Chemical analyses of fat, moisture, protein, ash, syneresis, acidity, pH, sodium, magnesium, and calcium contents were conducted. Instrumental texture analysis using the back extrusion method was applied. Quantitative descriptive sensory analysis was used to profile samples by 11 trained panelists and the acceptability of samples was assessed by 47 panelists. Type of milk significantly affected (*P* < 0.001) all chemical attributes except moisture and nitrogen-free extract, and fat level significantly impacted moisture, fat, protein, ash, acidity, and magnesium contents of Labneh. Type of milk significantly affected apparent modulus, hardness, hardness work done, and adhesive force, whereas fat level significantly affected hardness. Type of milk significantly affected the sensory attributes of syneresis, compactness, goaty odor and flavor, rate of flow, color, shininess, bitter flavor, denseness, melting rate, and spreadability, whereas fat level affected only color, denseness, and melting rate. Type of milk had a significant effect on overall acceptability and acceptability of flavor and texture.

## Introduction

Public attention has been recently directed toward the issue of diet and health. Increasing concern over the epidemics of obesity, coronary heart disease, hypertension, and cardiovascular diseases has oriented consumers for searching for food products that are lower in fat content (Alonso et al. 2009). Dietary saturated fat has been labeled as one of the major causes of the above-mentioned diseases. As milk contains saturated fatty acids and cholesterol, some researchers consider full-fat milk and its products as one of the food items that could promote a higher intake of saturated fat. Consequently, consumer perception has resulted in higher consumption of low-fat foods. In fact, Canadian dairy consumption patterns have been strictly affected by the amounts of dietary fats (Cash et al. 2005). Consumer preference for lower fat food has triggered companies to produce new brands of products to satisfy the needs of this market. However, decreasing fat in dairy products could be detrimental to the sensory and textural properties.

Concentrated/Greek yogurt or Labneh is a fermented dairy product produced by the process of elimination of whey from yogurt. This product, which originated in the Middle East, has found wide distribution all over the world due to its high nutritional benefits. Many studies have been conducted on the rheological, microbiological, compositional properties, and processing parameters affecting the production of concentrated yogurt (Salji et al. 1987; Mehaia and El-Khadragy 1999; Ozer et al. 1999; Abu-Jdayil et al. 2002). However, few studies have been conducted on the sensory properties of Labneh (Rao et al. 1987; Malek et al. 2001). Moreover, the effect of fat level, as exemplified in different types of milk, has not been thoroughly studied and its impact on the sensory properties of Labneh has not been elucidated. The objective of this work was to characterize the physicochemical and sensory properties of bovine, caprine, and ovine Labneh with different fat levels.

## Material and Methods

### Labneh production

Production of Labneh took place at the creamery of the American University of Beirut (AUB; AREC, Hosh Sneid, Lebanon). Cow, goat, and sheep milk collected from the university's farm at the end of April were standardized for fat level using Pearson's square method (Dairy Processing Handbook 1995). Fat and total solid levels of different types of Labneh were targeted to be consistent with the Lebanese Standards Institution (LIBNOR 1999) standards. Experimental samples were produced in two batches (2 days) at three fat levels (full-fat, reduced-fat, and low-fat) for three types of milk (bovine, caprine, and ovine) with a total of 18 samples. For each batch, 15 kg of recombined skim milk and cream were prepared to yield milk of varying fat contents (0.1–3.5%) to produce low-(LF), reduced-(RF) and full-fat (FF) bovine, caprine, or ovine milk Labneh (BL, CL, and OL, respectively), as described by Tamime and Robinson (2007) except that the pH of fermentation was reduced to 4.2. Fat standardized milk was pasteurized at 85°C for 30 min in a 100-L vat. After incubation at 42°C, and prior cooling in a 4°C refrigerator to reach the incubation temperature, 30 mL of CH1 culture solution (Chr. Hansen laboratories, Hørsholm, Denmark) was added as indicated by the culture manufacturer. Samples were incubated at 42°C, while covered, until a pH dropdown to 4.2 was achieved, and then they were transferred overnight to a walk-in cooler set at 2°C. Salt at a rate of 1% was added to every 15 kg of yogurt; this mixture was distributed into two bags and hung in a cooler to allow for whey drainage. Samples were hung for 18–50 h depending on the type of milk and fat level until a solid content of ∼26% (w/w) was obtained. Samples were filled and packed in 1 kg plastic food grade containers after minor gentle manual stirring. Total solid standardization was sometimes performed by mixing the required amount of whey with the proper amount of concentrated yogurt. Prepared samples were stored at 4°C until further analysis.

### Chemical analyses

Representative Labneh samples were sampled as indicated by AOAC (920.122, 955.30). Total solids, protein, fat in milk, fat in Labneh, ash, titratable acidity, and pH were determined by the AOAC 2005 official methods (926.08, 2001.14, 2000.18, 933.05, 935.42, and 920.124, respectively). Syneresis was determined as indicated by Al-Kadamany et al. (2003) but using Whatman No. 1 filter paper. Mineral analysis was performed by the acid digestion method according to the protocol mentioned for cheese ashes by the microwave Ethos Plus (Milestone Inc., Monroe, CT), June 2000.

### Instrumental texture analysis

Textural properties of Labneh were evaluated instrumentally using a texture analyzer (Brookfield QTS25; Brookfield Engineering Labs, Middleboro, MA), as described in Kaaki et al. (2012). Speed of cross-head was 10 mm/min and parameters measured were adhesiveness, adhesive force, apparent modulus, hardness, and hardness work done. Measurements were conducted in triplicate.

### Descriptive analysis

Eleven panelists (nine women and two men, mean age = 25) were recruited based on their willingness to participate and their consumption of Labneh on a regular basis. Descriptive analysis was conducted as described for Labneh in Kaaki et al. (2012). Six 1 h training sessions were conducted. The samples that were evaluated were gently stirred to homogenize them, filled in 30 oz. plastic containers, covered, and stored in the refrigerator at 4°C until served to the panelists. Samples were coded with three-digit random numbers. Table 1 summarizes the final list of 25 attributes along with definitions, anchor words, and standards used. Order of presentation of samples was counterbalanced based on the design for 18 samples (MacFie and Bratchell 1989). For every sample, two containers were filled with ∼35 g of Labneh for evaluation of most attributes and with 20 g for evaluation of syneresis, evenness of surface, stickiness, rate of flow, and spreadability. Evaluation of all samples was conducted in duplicate (3 milk types × 3 fat levels × 2 batches × 2 replicates). Serving of samples to panelists was conducted in three and a half days, two sessions per day (five samples/session) with one session on the fourth day (six samples). A minimum of 2 h was required between every two consecutive sessions. Descriptive evaluation of the samples took place in separated booths with fluorescent lighting in the sensory evaluation laboratory.

**Table 1:** Terms used in descriptive analysis of bovine, caprine, and ovine Labneh.

Attribute	Definition as worded on score sheet
Color	Color of Labneh ranging from chalky white to light ivory
Evenness of color	Presence of areas of different colors or shades of color on the surface of the sample
Syneresis	Amount of water that surrounds the mass of the sample
Evenness of surface	Appearance of granules or bumpy surface after flattening the sample with the spoon
Compactness	Extent by which the sample appears to be cohesive and firm visually
Shininess	Amount of light reflected from the surface of the sample
Sour/fermented odor	Sour odor elicited by goat Labneh
Milky odor	Odor elicited by whipping cream
Goaty odor	Odor elicited by goat milk
Saltiness	Taste elicited by table salt
Sour/fermented flavor	Sour taste elicited by goat Labneh
Milky flavor	Flavor elicited by whipping cream
Goaty flavor	Flavor of goat milk
Bitterness	Taste elicited by cold concentrated tea
Lumpiness	Feeling of lumps in the mouth upon pressing the sample between the palate and tongue
Denseness	Denseness of sample when pressed between tongue and palate
Water content	Amount of water that flows from the sample after pressing it in the mouth between palate and tongue
Melting rate	Rate at which the sample dissolves in the mouth
Stickiness	Degree by which the sample sticks to the back of spoon upon pressing the sample with the spoon and pulling it
Rate of flow	Rate with which the sample pours when 1/4 of a spoon is filled with sample and tilted
Spreadability	Ease of spreading a spoonful of sample with the spoon on a cracker
Residual film	Layer that remains on the palate after swallowing the sample
Salty aftertaste	Taste elicited by table salt in the mouth after swallowing the sample
Sour aftertaste	Sour taste elicited by goat Labneh standard in the mouth after swallowing the sample
Astringency	Shrinking or puckering and/or dryness of the tongue and palate

### Hedonic evaluation

An acceptability test was conducted with 47 panelists (22 men and 25 women, mean age = 27 years) in a similar manner to Kaaki et al. (2012) but with 18 samples instead of 17. Samples were served as six per session over two consecutive days. Panelists were asked to rate samples for appearance, flavor, texture, and overall acceptability using the 9-point hedonic scale (from 1 = dislike extremely to 9 = like extremely).

### Statistical analyses

PROC Mixed of SAS® (version 8.02, 1999–2001; SAS, Cary, NC) was used to perform the analysis of variance in a similar manner to Kaaki et al. (2012). Factors in model were milk type (bovine, ovine and caprine), fat level (FF, reduced fat or low fat), panelist, batch (nested within milk type and fat level), replicate (nested within batch), and their two-way interactions. Panelist and batch were included as random effects and milk type, fat level, and replicate were fixed effects in the model. Panelist was not included in chemical or physical analyses models and the sensory acceptability model did not include replicate. Significant means for sensory analyses were separated by Tukey's honestly significant difference. Significance was preestablished at *α *< 0.05. Principal component analysis was performed using the 36 means (3 types of milk × 3 fat levels × 2 batches × 2 replicates) obtained from descriptive analysis. In each part of the results and discussion section (chemical analyses, descriptive analysis, etc.), the first part summarizes the significant effects (*P*-values) of the main factors and their interactions but these results are not reported in tables. Mean comparisons are then discussed in text and reported in Tables 2,3,5 and 6. This was done to save on space/redundancy and to minimize the number of tables.

## Results and Discussion

### Physical and chemical analyses

Type of milk significantly affected (*P* < 0.001) protein, fat, ash, calcium, magnesium, and acidity. Moreover, nitrogen-free extract (NFE), syneresis (*P* < 0.01), and pH (*P* < 0.05) were significantly affected by type of milk. Fat level significantly affected (*P* < 0.001) moisture, fat, protein, ash, acidity (*P* < 0.01), and magnesium (*P* < 0.05). No significant type of milk × fat level interaction was recorded except for fat content. Least mean squares of chemical analyses variables are listed in Table 2. The moisture content of different milk type samples did not vary significantly. The fat content of bovine and caprine Labneh (BL and CL) was significantly lower than that of ovine Labneh (OL) samples; however, CL fat content did not vary significantly from BL fat content. Protein and magnesium contents of BL were significantly lower than CL and OL with no significant difference between the last two. Similar CL protein composition was reported by Mehaia and El-Khadragy (1999) where yogurt was concentrated to 23% total solid level. Malek et al. (2001) found no significant protein differences among different types of Labneh produced. However, because proteins are complex molecules that are highly retained during straining of yogurt, as reported by Tamime et al. (1991), and as sheep milk is typically higher in protein concentration compared to cow milk (Jandal 1996), it is reasonable to anticipate higher protein content in OL compared to BL. In this work, BL and CL samples showed no significant differences in ash content although these had significantly lower ash content than OL. OL also turned out to have the highest amount of calcium content with BL being the lowest. Rao et al. (1987) reported significantly higher results of calcium content in CL than in BL. The calcium content of sheep milk was reported by Jandal (1996) to be higher than goat milk followed by cow milk. No differences in sodium content were noted in this work. Güler and Şanal (2009) found significantly higher sodium content in sheep torba yogurt than cow torba yogurt. However, their results were much lower due to the absence of salt addition during production. Rao et al. (1987) reported no significant sodium difference between BL and CL. As for the magnesium contents of BL in this study, they were significantly lower than CL and OL with no significant difference between the last two. BL had a significantly higher amount of NFE than CL but not significantly different from OL, which was also the case for the last two. Malek et al. (2001) found no significant differences in lactose levels. The variation in lactose or NFE levels from the literature could be attributed to cloth type used and to original lactose levels in milk. BL exhibited significantly higher levels of syneresis than CL and OL with no difference in the last two. Acidity levels have been associated with degree of syneresis in acid milk gels (Van Vliet et al. 1997). However, even though no significant acidity differences were detected between BL and CL, syneresis differences existed. Syneresis has been attributed to structural rearrangements in the casein gel network (Van Vliet and Walstra 1994) and to the void space volume of the protein matrix (Tamime et al. 1991) and since protein is a basic element of the gel network, consequently, syneresis differences in this work could be attributed to protein content differences. The higher the protein content, the more complex is the gel network and the higher is the capacity of whey-holding void spaces. However, this factor requires further study to determine its effect on syneresis. No significant difference was obtained between pH of BL and CL samples whereas the pH of OL was significantly higher than BL although not significantly different than CL. In the case of acidity, BL and CL samples were significantly lower than OL. Güler and Şanal (2009) reported the acidity of torba yogurts of sheep, goat, and cow as 2.1%, 2.0%, and 1.8%, respectively, but with no significant differences. Although the trend of their data was similar to this work, their results were somehow higher. A possible factor for lower acidity in this work was the amount of concentration of total solids, because the higher concentration of solids with an average of 26% (w/w) in this work could have led to higher lactic acid levels.

**Table 2:** Least squares means of the chemical properties of Labneh samples for milk type (bovine, caprine, and ovine) and fat level (full fat, ∼10%; reduced fat, ∼5%; and low fat, <1%).

Chemical analyses	Type of milk			Fat level		
	Bovine	Caprine	Ovine	Full fat	Reduced fat	Low fat
Moisture	76.81^a^	77.25^a^	76.68^a^	73.88^a^	76.86^b^	80.01^c^
Fat	4.46^a^	4.61^a^	5.24^b^	9.18^a^	4.79^b^	0.35^c^
Protein	8.40^a^	10.00^b^	9.69^b^	8.14^a^	9.30^b^	10.65^c^
NFE	7.92^a^	5.52^b^	6.85^ab^	6.36^a^	7.44^a^	6.48^a^
Ash	1.50^a^	1.56^a^	1.74^b^	1.56^a^	1.58^a^	1.66^b^
Sodium	167.77^a^	175.74^a^	202.83^a^	158.92^a^	183.40^a^	204.00^a^
Calcium	0.25^a^	0.31^b^	0.37^c^	0.30^a^	0.30^a^	0.32^a^
Magnesium	3.70^a^	4.81^b^	4.88^b^	4.05^a^	4.57^ab^	4.78^b^
Syneresis	51.12^a^	41.94^b^	41.87^b^	45.06^a^	46.85^a^	43.02^a^
pH	3.68^a^	3.75^ab^	3.81^b^	3.76^a^	3.73^a^	3.74^a^
Acidity	1.10^a^	1.15^a^	1.28^b^	1.12^a^	1.19^b^	1.22^b^

Moisture, fat, protein, lactose, ash, calcium, and acidity are measured in percentage, whereas sodium and magnesium are in mg/100 g. NFE, nitrogen-free extract, by calculation. Means in a row with different alphabetical superscripts are significantly different (*P* < 0.05).

Moisture and fat content of different fat levels were significantly different between fat levels and were within targeted LIBNOR standards for different fat level Labnehs. Protein content of LF Labneh was the highest whereas that of FF samples was the lowest. Higher protein levels with LF samples were reasonably justified by the loss of fat that had to be replaced by another solid and/or by an increase in moisture content. As NFE levels were not significantly different among fat levels, it was logical to observe this increase in solids for the protein level. No significant differences in NFE, sodium, calcium, syneresis, and pH were noted among fat levels. The amount of ash in LF samples was significantly higher than FF and RF samples. Moreover, LF Labneh was significantly higher in magnesium than FF Labneh. The acidity of RF and LF samples, although not significantly different, was significantly higher than that of FF samples. Yazici and Akgun (2004) reported no significant differences in acidity levels of strained yogurt samples prepared with 0.5% and 2% milk fat. Upon examination of the means, LF samples did not differ significantly from each other in fat content, although they differed from RF and FF samples, which in turn significantly differed from each other.

### Instrumental texture analysis

The type of milk significantly affected apparent modulus, hardness, hardness work done (*P* < 0.001), and adhesive force (*P* < 0.01). Significant differences were obtained for fat levels with hardness (*P* < 0.05). No significant type of milk × fat level interaction was noted. Table 3 summarizes the least mean squares of texture analysis attributes. BL samples were leftacterized with significantly higher adhesive force, hardness, and hardness work done in comparison with CL and OL samples, which did not vary significantly from each other. Tamime and Robinson (1999) indicated that the firmness of BL was much higher than that of CL and OL. The apparent modulus of CL was significantly higher than that of BL and OL samples. No significant differences were noted with fat level except for hardness. LF samples were significantly harder than FF but with no difference with RF samples. Similar results were reported by Yazici and Akgun (2004) who found strained yogurts made with 0.5% milk fat to be significantly harder than samples made with 2.0% fat.

**Table 3:** Least squares means of the textural properties of Labneh samples for milk type (bovine, caprine, and ovine) and fat level (full fat, ∼10%; reduced fat, ∼5%; and low fat, <1%).

Textural analyses	Type of milk			Fat level		
	Bovine	Caprine	Ovine	Full fat	Reduced fat	Low fat
Apparent modulus	248.56^a^	810.50^b^	375.33^a^	437.89^a^	548.06^a^	448.43^a^
Adhesiveness	−770.47^a^	−689.58^a^	−557.50^a^	−567.45^a^	−747.80^a^	−702.30^a^
Adhesive force	−141.31^a^	−101.26^b^	−89.63^b^	−95.27^a^	−116.53^a^	−120.41^a^
Hardness	214.50^a^	130.08^b^	98.45^b^	120.93^a^	156.85^ab^	167.26^b^
Hardness work done	5135.50^a^	3159.68^b^	2209.48^b^	2714.33^a^	3828.21^a^	3962.12^a^

Hardness and adhesive force are measured in grams (g) whereas apparent modulus, adhesiveness, and hardness work done are measured in gram seconds (g.sec). Means in a row with different alphabetical superscripts are significantly different (*P* < 0.05).

### Descriptive analysis

#### ANOVA and Tukey's test

Significant effects and least mean squares of descriptive attributes are summarized in Tables 4,5 respectively. There were significant differences between types of milk for syneresis, compactness, goaty odor, goaty flavor, rate of flow (*P* < 0.01), color, shininess, bitter flavor, denseness, melting rate, and spreadability (*P* < 0.05). On the other hand, fat level only affected color (*P* < 0.01), denseness, and melting rate (*P* < 0.05).

**Table 4:** Significance of the effects of type of milk (bovine vs. caprine vs. ovine), fat level (full fat, ∼10%; reduced fat, ∼5%; and low fat, <1%), and their interactions on the descriptive attributes of Labneh samples.

Descriptive attributes	Effect *F* values					
	Type df = 2	Fat level df = 2	Replicate df = 3	FL × T df = 4	FL × Replicate df = 6	T × Replicate df = 6
Color	9.66*	26.06**	1.08	12.09*	0.67	1.46
Evenness of color	1.91	1.41	0.70	0.87	0.96	0.88
Syneresis	55.01**	1.47	1.46	0.81	2.60*	2.32*
Evenness of surface	1.81	5.16	0.79	0.26	0.71	0.34
Compactness	18.97**	0.51	0.46	0.69	2.14	2.04
Shininess	7.89*	5.32	0.01	1.81	0.67	0.32
Sour-fermented odor	0.63	2.14	2.20	0.46	1.19	1.63
Milky odor	0.51	0.08	0.27	0.24	0.25	0.68
Goaty odor	20.29**	1.98	5.50	1.05	0.35	0.76
Salty flavor	2.89	0.38	6.11	1.26	0.81	0.90
Sour flavor	1.90	3.21	0.61	0.12	0.48	0.61
Milky flavor	0.61	0.23	0.37	0.61	0.18	2.10
Goaty flavor	32.00**	0.25	5.15**	3.73	1.03	1.06
Bitter flavor	10.28*	4.14	1.62	1.93	0.64	1.69
Lumpiness	0.02	3.63	0.00	1.85	2.95**	1.00
Denseness	16.47*	15.81*	0.14	1.36	3.32**	2.00
Water content	6.40	6.62	0.07	3.33	1.23	2.84*
Melting rate	17.13*	8.36*	0.15	1.68	1.26	3.41**
Rate of flow	23.75**	4.45	0.69	1.02	1.52	0.92
Stickiness	0.66	0.64	2.42	0.22	0.30	0.56
Spreadability	8.32*	0.57	0.74	0.39	0.81	0.22
Residual film	1.27	1.57	0.46	0.55	1.83	1.09
Salty aftertaste	2.99	0.77	3.06	1.12	1.44	0.93
Sour aftertaste	0.09	1.31	2.13	0.89	0.31	0.69
Astringency	3.74	3.24	0.19	2.37	0.28	1.25

FL, fat level; Rep, replicate; T, type.

* *P* < 0.05;

** *P* < 0.01.

**Table 5:** Least squares means of the descriptive attributes of Labneh samples for milk type (bovine, caprine, and ovine) and fat level (full fat, ∼10%; reduced fat, ∼5%; and low fat, <1%).

Descriptive analyses	Type			Fat level		
	Bovine	Caprine	Ovine	Full fat	Reduced fat	Low fat
Color	7.20^a^	5.01^b^	6.21^ab^	7.41^a^	5.92^b^	5.09^b^
Evenness of color	10.83	11.70	11.43	11.02	11.33	11.43
Syneresis	11.95^a^	4.44^b^	4.38^b^	6.34	7.60	6.83
Evenness of surface	8.44	9.99	9.67	8.06	9.12	10.91
Compactness	8.80^a^	4.35^b^	4.15^b^	5.64	5.97	5.70
Shininess	8.19^a^	10.33^b^	9.95^ab^	8.91	9.61	9.47
Sour-fermented odor	9.41	9.03	9.04	8.78	9.29	9.40
Milky odor	7.14	7.52	7.06	7.31	7.15	7.26
Goaty odor	4.28^a^	8.77^b^	8.56^b^	6.92	6.92	7.77
Salty flavor	7.85	8.20	8.62	8.04	8.27	8.37
Sour flavor	8.39	9.18	8.85	8.40	8.88	9.15
Milky flavor	6.85	6.43	6.88	6.85	6.66	6.66
Goaty flavor	4.14^a^	10.37^b^	9.14^b^	7.78	7.82	8.05
Bitter flavor	4.58^a^	7.03^b^	6.78^b^	5.56	6.37	6.47
Lumpiness	4.22	4.32	4.31	5.09	4.38	3.38
Denseness	7.24^a^	4.94^b^	4.33^b^	4.73^a^	5.55^ab^	6.23^b^
Water content	7.70	9.04	9.83	9.53	8.55	8.47
Melting rate	8.29^a^	10.40^b^	10.51^b^	10.42^a^	9.69^ab^	9.10^b^
Rate of flow	5.78^a^	9.27^b^	10.05^b^	9.41	8.22	7.46
Stickiness	8.17	7.80	7.22	7.34	7.87	7.98
Spreadability	9.35^a^	11.15^ab^	11.20^b^	10.80	10.58	10.32
Residual film	7.37	6.64	6.74	6.49	6.98	7.27
Salty aftertaste	6.90	7.33	7.60	7.41	7.07	7.35
Sour aftertaste	8.72	8.87	8.83	8.65	8.72	9.06
Astringency	6.34	8.11	7.86	7.14	7.29	7.88

Means in a row with different alphabetical superscripts are significantly different (*P* < 0.05).

BL had significantly more light ivory color than CL which was whiter in color. Malek et al. (2001) reported BL to have a significantly yellowish tint in comparison with the white color of OL and CL due to higher amounts of carotene found in bovine milk. CL samples showed a significantly shinier appearance than BL samples. BL samples were significantly more compact in appearance, denser in mouthfeel, and exhibited higher levels of visual whey syneresis than CL and OL samples that did not differ significantly in these attributes. Malek et al. (2001) reported similar trends for these attributes. BL samples were described to be thicker and more cohesive than OL and CL samples. In fact, sensory denseness of samples highly correlated with instrumental hardness (*R*^2^ = 0.90, *P* < 0.05). Sensory measurements in syneresis also coincided well with physical measurements (*R*^2^ = 0.79, *P* < 0.05), whereby BL showed higher levels of syneresis than OL and CL samples. BL had significantly less goaty odor and goaty flavor, less bitterness, slower melting rate, and slower rate of flow than CL and OL samples that did not differ significantly. The fact that no significant differences were obtained for goaty odor and flavor between CL and OL could be due to lack of familiarity of sensory judges with these two products despite the extensive training with leftacteristic flavors of goat and sheep milk. These flavors were described by Ha and Lindsay (1993) as mutton-like and goat-like flavors. The ease of spreadability of OL was significantly higher than BL. The opposite was obtained for flow and melting rates where BL had significantly lower ratings than CL and OL, in a similar manner to Malek et al. (2001), who attributed the above differences to the structural strength of Labneh. BL samples were leftacterized with a compact and uniform microstructure with larger and irregular void spaces than OL and CL, and BL was able to resist more stress than CL and OL (Tamime et al. 1991). On the other hand, sensory stickiness highly correlated with instrumental adhesive force (*R*^2^ = −0.76, *P* < 0.05), unlike sourness and acidity as measured by titrimetric methods (*R*^2^ = 0.41, *P* > 0.05).

Low fat content showed a significantly denser and a slower melting rate of samples compared to FF or RF Labneh, with no significant differences noted between the RF and the two other fat level samples.

A significant replicate effect (*P* < 0.01) appeared only for goaty flavor. A significant type of milk × fat level interaction was observed only for color (*P* < 0.05). FF-BL was the only product with significantly higher ivory color compared to all other products (with a rating of 9.82^a^). However, RF-BL (7.30^ab^), FF-OL (6.73^ab^), and LF-OL (6.73^ab^) were not significantly different from FF-BL, which removed any trend in the response of the panelists. Yazici and Akgun (2004) reported a significant increase in whiteness values of concentrated yogurt with lower fat content.

Significant fat levels × replicate interactions were noted for syneresis (*P* < 0.05), lumpiness, and denseness (*P* < 0.01). Moreover, type × replicate interactions were obtained for syneresis (*P* < 0.05), water content (*P* < 0.05), and melting rate (*P* < 0.01). No major inconsistencies in the panelists' ratings appeared as shown by the absence of replicate effect and of significant interactions for most attributes.

#### Principal component analysis

**Figure 1:**
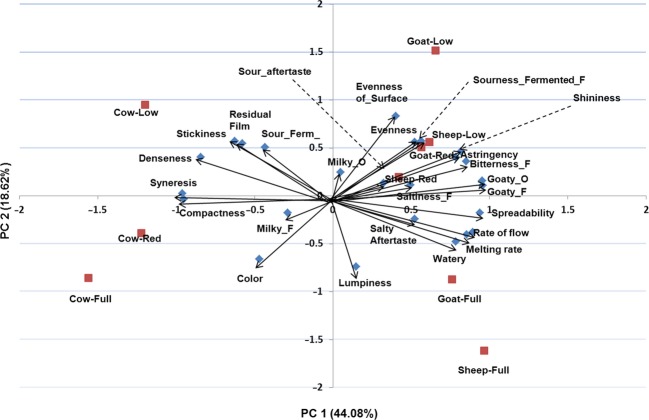
Principal component plot of Labneh samples and attributes. Cow-Full, full-fat bovine Labneh; Goat-Full, full-fat caprine Labneh; Sheep-Full, full-fat ovine Labneh; Cow-Red, reduced-fat bovine Labneh; Goat-Red, reduced-fat caprine Labneh; Sheep-Red, reduced-fat ovine Labneh; Cow-Low, low-fat bovine Labneh; Goat-Low, low-fat caprine Labneh; Sheep-Low, low-fat ovine Labneh.

Figure 1 illustrates the first two principal components (PC), which accounted for 62.7% of the variation with 44.08% and 18.62% by PC 1 and PC2, respectively. PC 1, which is displayed horizontally on the scale, separated attributes based on type of milk. The positive cluster (right-hand side) of PC1 included attributes that are highly descriptive of CL and OL whereas the negative cluster of attributes had attributes that mainly leftacterized BL. Thus, attributes such as goaty odor and flavor, bitterness, rate of flow, melting rate, etc. that were given high scores by panelists (Table 5) for CL and OL were included on the positive side of PC1, unlike BL, which clustered on the negative side of PC1. The lower ratings for FF-BL on rate of flow and melting rate, despite the lack of significant differences on moisture and fat for FF-BL, FF-CL, and FF-OL, might be due to the different fat composition and structure of these different types of milk products as noted above. PC2 separated attributes based on the fat level. The upper right and left clusters represent the positive side of PC2 and vice versa. Thus, the positive side of PC2 combined attributes highly descriptive of LF and RF samples such as LF-BL, LF-CL, LF-OL, RF-CL, and RF-OL samples. Attributes such as stickiness, residual film, sour-fermented odor, etc., leftacteristic of LF and RF samples, were clustered on the positive side of PC2. Attributes highly scored for FF-BL, FF-OL, and FF-CL and RF-BL were included on the negative side of PC2. It is notable that RF-BL was included on the negative part of PC2; however, it was on the border part of PC2, indicating that it lay in the middle of the LF and FF clusters. The same applied to the RF-OL sample.

### Hedonic evaluation

Least means squares of acceptability parameters are summarized in Table 6. Type of milk significantly affected overall acceptability, flavor (*P* < 0.01), texture, and appearance (*P* < 0.05). No significant fat level effect or fat level × type interaction was noted. Overall acceptability, acceptability of texture, and flavor were significantly higher for BL than for OL and CL. The appearance acceptability of BL did not differ significantly from OL, although it was significantly higher than that of CL. No significant differences in acceptability parameters were observed between CL and OL samples. Similar results in acceptability of BL, OL, and CL were reported by Malek et al. (2001), where BL had significantly higher acceptability ratings than CL and OL, which did not differ. Low acceptability of OL and CL was attributed to the sharp caprine flavor, which was objectionable to the panelists.

**Table 6:** Least squares means of the acceptability parameters of Labneh samples for milk type (bovine, caprine, and ovine) and fat level (full fat, ∼10%; reduced fat, ∼5%; and low fat, <1%).

Acceptability	Type of milk			Fat level		
	Bovine	Caprine	Ovine	Full fat	Reduced fat	Low fat
Overall acceptability	5.96^a^	3.65^b^	4.44^b^	4.70	4.74	4.61
Appearance	6.17^a^	5.11^b^	5.33^ab^	5.52	5.45	5.63
Texture	6.28^a^	4.89^b^	5.04^b^	5.36	5.32	5.53
Flavor	5.80^a^	3.32^b^	4.11^b^	4.54	4.46	4.24

Means in a row with different alphabetical superscripts are significantly different (*P* < 0.05).

**Figure 2:**
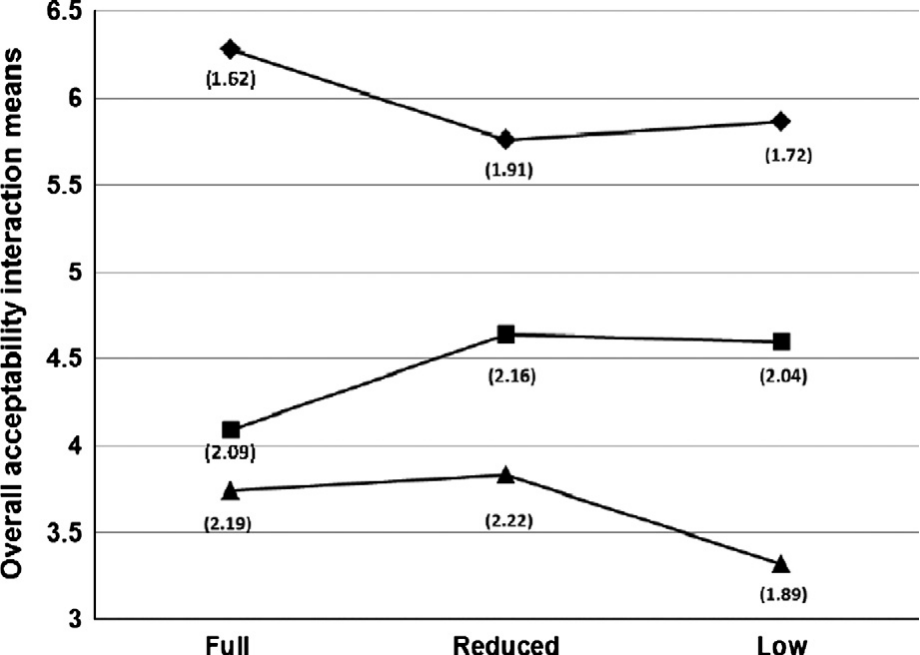
Overall acceptability means of full (∼10%), reduced (∼5%); and low fat (<1%) bovine (♦), caprine (▴), and ovine (▪) Labneh samples. Numbers in parenthesis indicate the SD of the means.

The lack of differences between fat levels was rather surprising, especially given that previous work had shown differences between FF-BL and LF-BL samples (Kaaki et al. 2012), and could be due to the lack of major differences in texture of varying fat level Labneh samples which is not typical of products such as cheese and meat where these differences could be easily noticed by naïve consumer panelists, or it could be an artifact of the statistical analyses with different variables and levels. Lack of differences could also be due to the small amount of sample assessed and to overwhelming types of milk differences, which could have overshadowed fat level differences. Figure 2 shows that the overall acceptability of FF-BL was relatively rated higher than those of LF samples. However, the case is reversed in OL samples, a phenomenon that could be partly attributed to carry-over effect, although the panel administrators made sure that panelists rinsed between samples. In analyzing the carry-over effect of restructured steak, Schlich (1993) reported that out of 15 attributes studied, five showed some evidence of carry-over. On the other hand, Ferris et al. (2003) suggested that special attention must be paid in sensory trial designs when consumer survey preference tests are conducted since the carry-over effect is more likely to take place when the assessed number of attributes in a trial is small. Of course, another highly likely possibility for the low acceptability of the FF-OL was the type of fat in OL, which gave it a very soft, if not “slimy,” texture not typical of the regularly consumed BL and thus a decrease in fat content could have resulted in a more typical texture and thus a higher acceptability level. The mutton-like flavor, highly concentrated in the fatty part of the FF-OL, could have also contributed to the above result.

## Conclusion

The production of Labneh with different milk types, especially with different fat levels, is highly economical and required by the low-fat product-oriented market. The type of milk significantly affected (*P* < 0.001) all chemical attributes but moisture and NFE, and fat level significantly affected moisture, fat, protein, ash, acidity, and magnesium of Labneh. The type of milk significantly affected apparent modulus, hardness, hardness work done, and adhesive force, whereas fat level significantly affected hardness and hardness work done. The type of milk significantly affected the sensory attributes of syneresis, compactness, goaty odor, and flavor, rate of flow, color, shininess, bitter flavor, denseness, melting rate, and spreadability, whereas fat level affected only color, denseness, and melting rate. The type of milk had a significant effect on the overall acceptability and acceptability of flavor and texture.

This study has shown that consumer perception of sensory parameters is highly sensitive to sensory attributes such as goaty and sheep flavors**.** The results of this study, although have derived a sensory lexicon for different types of Labneh with different fat levels, have highlighted the need to implement several modifications. Labneh production should be preferably performed during a specific lactation period whereby the goaty odor and flavor may be modulated differently. The adopted fat levels may have produced a high moisture content in ovine samples which, although following LIBNOR standards, have shown to be too liquidy and thus could have affected sensory results. The differences noted in this study should be helpful to dairy companies for accommodating changes and effects of type of milk and fat level on Labneh products so as to increase the marketability and acceptability of this product while satisfying market demands.

Finally, a disadvantage of fat reduction was that LF samples were significantly harder than FF but showed no difference with RF samples. However, this did not translate into acceptability differences between FF-BL and RF-BL, which is advantageous to the marketing of RF-BL and FF-BL and has been gaining a higher market share over the years.
